# The Retraction

**Published:** 1851-08

**Authors:** 


### THE RETRACTION.

Under circumstances that seemed proper at the time, I had voluntarily resolved to make a retraction of one word in my response o Dr. Bullitt, in the July number of this Journal, and that resolution was formed before the number was ready for delivery. In a conversation with one of Dr. Bullitt's friends, I stated the fact of my intention, and subsequently he requested of me a declaration of my intentions so that he could communicate them to Dr. Bullitt. In order to avoid errors, I drew up an explanatory note on the subject, and gave it to the gentleman. But having learned that a very improper view has been taken of this note, and that it is used contrary to the purposes designed, I sought the gentleman to whom the note was given, and stated to him that I should make no retraction of the term "deliberate misrepresentation" contained in my response to Dr. Bullitt's assault, but should let it remain as it stood in my response. B.

